# Melatonin Priming Alleviates Aging-Induced Germination Inhibition by Regulating β-oxidation, Protein Translation, and Antioxidant Metabolism in Oat (*Avena sativa* L.) Seeds

**DOI:** 10.3390/ijms21051898

**Published:** 2020-03-10

**Authors:** Huifang Yan, Shangang Jia, Peisheng Mao

**Affiliations:** 1 Forage Seed Laboratory, China Agricultural University, Beijing 100193, China; yanhuifang@qau.edu.cn (H.Y.); shangang.jia@cau.edu.cn (S.J.); 2 Grassland Agri-husbandry Research Center, College of Grassland Science, Qingdao Agricultural University, Qingdao 266109, China; 3 Key Laboratory of Pratacultural Science, Beijing Municipality, China Agricultural University, Beijing 100193, China

**Keywords:** melatonin, seed aging, germination, oat, β-oxidation, protein translation, antioxidant defense

## Abstract

Although melatonin has been reported to play an important role in regulating metabolic events under adverse stresses, its underlying mechanisms on germination in aged seeds remain unclear. This study was conducted to investigate the effect of melatonin priming (MP) on embryos of aged oat seeds in relation to germination, ultrastructural changes, antioxidant responses, and protein profiles. Proteomic analysis revealed, in total, 402 differentially expressed proteins (DEPs) in normal, aged, and aged + MP embryos. The downregulated DEPs in aged embryos were enriched in sucrose metabolism, glycolysis, β-oxidation of lipid, and protein synthesis. MP (200 μM) turned four downregulated DEPs into upregulated DEPs, among which, especially 3-ketoacyl-CoA thiolase-like protein (KATLP) involved in the β-oxidation pathway played a key role in maintaining TCA cycle stability and providing more energy for protein translation. Furthermore, it was found that MP enhanced antioxidant capacity in the ascorbate-glutathione (AsA-GSH) system, declined reactive oxygen species (ROS), and improved cell ultrastructure. These results indicated that the impaired germination and seedling growth of aged seeds could be rescued to a certain level by melatonin, predominantly depending on β-oxidation, protein translation, and antioxidant protection of AsA-GSH. This work reveals new insights into melatonin-mediated mechanisms from protein profiles that occur in embryos of oat seeds processed by both aging and priming.

## 1. Introduction

Seeds are the genetic materials for cultivation of almost all crop species in agriculture. Seed germination represents the most crucial initiation for crop development and growth, greatly relying on seed physiological quality, and influences the subsequent seedling performances in various environments [1]. However, as storage duration is extended, seed vigor gradually decreases and aging inevitably occurs; as a result, seeds germinate poorly and non-uniformly, and economic and genetic losses are caused [2]. The auto-oxidative reactions and resulting accumulation of reactive oxygen species (ROS) are considered as the key factors underlying seed aging, which lead to diverse deleterious metabolic alterations including disruption of cellular membranes, degradation of nucleic acid, and damage of lipids and proteins [3]. Proteomic studies on the embryo of artificially aged wheat (*Triticum aestivum* L.) [4] and rice (*Oryza sativa* L.) [5] seeds showed that differentially expressed proteins (DEPs) were mainly involved in nutrient reservoir, enzyme activity and regulation, energy supply and metabolism, as well as defense and stress responses, which were consistent with the previous physiological and biochemical studies. Hitherto, multifarious practical strategies have been adopted to prevent or retard seed aging and vigor loss during storage, such as cryopreservation, ultra-dry storage, and free-oxygen environment [6,7,8]. However, for aged seeds, delayed germination and slow post-germination growth are still the two most restrictive problems impeding their application under field conditions [9]. Therefore, it is necessary to explore seed aging and germination mechanisms, and new strategies should be developed to improve the compromised vigor and seeding value of aged seeds.

Seed priming, a technique applied by imbibing seeds with water or various chemical reagents (e.g., vitamins, antioxidants, and hormones) to trigger the germination-related events before radicle protrusion, is widely used to reinforce vigor and enhance germination and emergence of aged seeds [10]. The improved germination characteristics are attributed to the activation of priming-induced metabolic activities, referred to as “priming memory”, including de novo synthesis of nucleic acids and proteins, ATP production, restoration of antioxidant activities, and cellular repair [11]. Previous studies about seed priming mainly focused on its benefits, involving germination features, antioxidant process, cell cycle and cell structures, and expression of genes and proteins, against various abiotic stresses including drought, heavy metals, and chilling [10,12,13]. Very few researches have payed attention to the effects of artificially aging and subsequent priming, but only at germination and seedling growth, antioxidant enzymes’ activities, and seed reservoirs in species, such as cucumber (*Cucumis sativa* L.) [14] and maize (*Zea maize* L.) [9]. However, key metabolic changes and molecular mechanisms to understand the relationship between seed aging and priming have not yet been thoroughly studied.

Proteomic approaches have successfully created new avenues to detect changes in various cellular processes, understand biological functions of individual proteins, and elucidate metabolic mechanisms during seed aging and priming [15]. Embryonic proteomics of wheat seeds showed that 162 DEPs which were mainly involved in metabolism, energy supply, and defense/stress were identified during artificial aging, and 531 DEPs related to energy supply, amino acids and fatty acid synthesis, as well as cell growth and division were recognized during seed priming [4]. Although the above study has revealed new insights into DEPs’ changes that occur during seed aging and priming, there is still not enough information on protein changes to decipher the mechanisms of priming’s effects on answering the detriments of seed aging.

Melatonin (N-acetyl-5-methoxytryptamine) is a pleiotropic molecule ubiquitously distributed in diverse kingdoms, covering bacteria, insects, animals, and plants [16]. Melatonin in plants has been reported to play important roles in regulating multiple cellular and physiological activities, including improvement of seed vigor and germination [17], alleviation of leaf senescence [18], acceleration of growth, flowering and development [19], and resistance to stress conditions such as heavy metal, drought, chilling, and salinity [20,21,22,23]. As an endogenous scavenger and antioxidant, melatonin can directly remove excess ROS based on its extremely strong scavenging capacity [24]. In addition, melatonin plays an indirect role by enhancing activities of antioxidant enzymes and related genes, and improving mitochondrial efficiency under environmental stresses [25,26]. Up-to-date, studies about melatonin’s role in metabolic events of aged seeds have been rarely reported. Recently, research on melatonin for alleviating aging-induced oxidative damage was reported by Su et al. [27] in maize seeds, which revealed that melatonin improved germination and growth characteristics, enhanced activities of antioxidases (superoxide dismutase (SOD), ascorbate peroxidase (APX), and catalase (CAT)), reduced lipid peroxidation (LPO), and induced various metabolic changes (e.g., hormone signal transduction, cellular processes, metabolism of carbohydrate, secondary metabolite, and amino acid) by physiological and transcriptome analysis. However, there are still limited data to elucidate the underlying mechanisms of melatonin priming (MP) response to seed aging.

Oat (*Avena sativa* L.) is an important cereal crop possessing high levels of valuable nutrients such as protein, unsaturated fatty acids, soluble dietary fiber and minerals [28], and it is widely utilized to provide nutritional consumption for human and livestock [29]. Due to their high oil content (up to 18% in specific cultivars) and the resulting oxidation rancidity of polyunsaturated fatty acid [30], oat seeds are prone to aging during storage, which has limited their widespread use and caused great economic losses [31]. Therefore, it is necessary and important to take a rejuvenated technique to improve the repressed vigor and germination in aged oat seeds.

This study was conducted to determine the ultrastructural, physiological, and proteomic changes in the embryo of oat seeds with aging process and the subsequent melatonin priming, including germination and seedling growth, cellular ultrastructure, ROS accumulation, and LPO, activities of antioxidant enzymes and DEPs’ information. The objective was to investigate whether the aging-induced negative impacts on oat seeds could be renovated by melatonin priming, and to illuminate the underlying mechanisms in response to MP in aged oat seeds.

## 2. Results

### 2.1. Germination and Subsequent Seedling Growth Characteristics under Aging and Melatonin Priming in Oat Seeds

The germination percentage (GP) of oat seeds showed a reverse “S-shaped” curve with prolonged aging duration (Figure S1A). Aging of 28 days (d) did not result in a significant difference in the GP (still 100%), while immediately after, it led to a decreased GP from 100% to 98% (32 d), 69% (48 d), 42% (56 d), and 0% (64 d). The GP declined rapidly from aging of 44 d (91%) to 52 d (44%), and no seeds germinated after being aged for 64 d at 45 °C. Therefore, on the basis of the reverse ”S-shaped” aging curve, the 48 d was selected for the aging process, with a moderate vigor level (approximately 70% GP).

**Figure 1:**
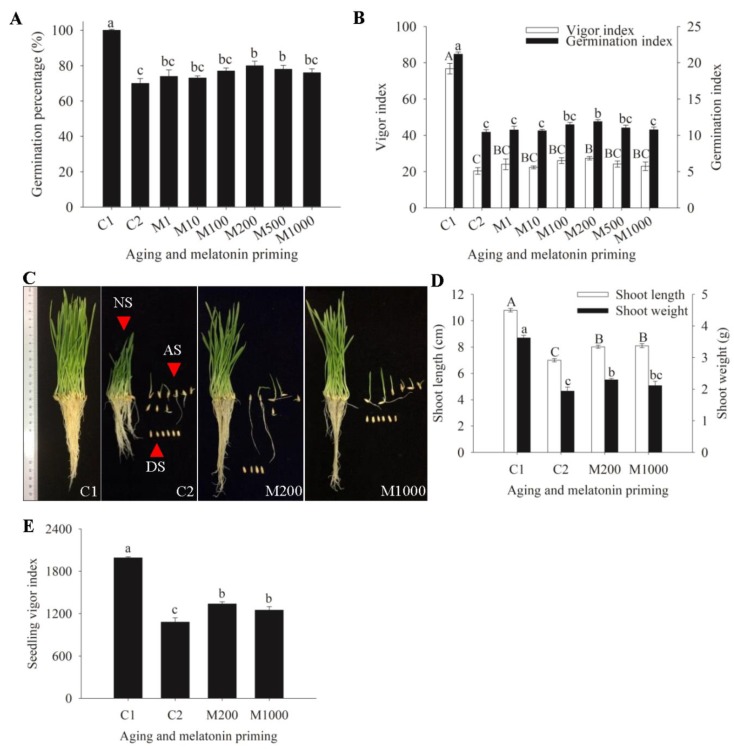
Seed germination and seedling development under aging and melatonin priming in oat. (**A**) Germination percentage (GP) calculated on the 10th day of germination. (C1) normal seeds, (C2) seeds with only 48-days aging treatment, and (M1~M1000) seeds with 48-days aging and further melatonin priming at concentrations of 1~1000 μM; (**B**) Seed vigor index (VI) and germination index (GI) calculated on the 10th day of germination; (**C**) The phenotype of seedling growth on the 10th day of germination. Normal seedlings (NS), abnormal seedlings (AS), and dead seeds (DS) were displayed in C2, M200, and M1000 on the left, the upper right, and the lower right, respectively; (**D**) Shoot length (SL) and shoot weight (SW) of seedlings at the 10th day of germination; (**E**) Seedling vigor index (SVI). Values were recorded by the means ± SE (*n* = 4). Different letters indicated significant differences of aging and melatonin priming at the *p* < 0.05 level.

The 48-days aging duration significantly decreased the GP from 100% (C1) to 70% (C2) in oat seeds, with similar changes shown in seed vigor index (VI) and germination index (GI) (Figure 1A,B). Priming treatment on aged oat seeds with melatonin slowed the aging-induced germinability decline. Although various concentrations of melatonin all had the alleviation effects on GP decline, this down trend could be significantly retarded with priming of 200 and 500 μM, i.e., M200 and M500 (Figure 1A). Furthermore, 200 μM melatonin significantly mitigated the decline of VI and GI (Figure 1B). On the basis of these screening results, 200 μM of melatonin could significantly improve germinability of aged seeds and was selected for further experiments. In addition, the high concentration of 1000 μM with no significant effect was also selected.

The phenotype of seedling development on the 10th day was repressed after 48-days aging duration at 45 °C, with more abnormal seedlings and dead seeds (Figure 1C). Meanwhile, shoot length (SL), shoot weight (SW), and seedling vigor index (SVI) were significantly reduced by 35.2%, 46.4%, and 45.6%, respectively (Figure 1D,E). However, MP obviously improved the phenotypic performances in seedling development of aged seeds, with less abnormal seedlings and dead seeds, especially at the level of 200 μM (Figure 1C), and SL, SW, and SVI at 200 μM were significantly increased by 14.6%, 18.4%, and 23.7%, respectively. Moreover, 1000 μM of melatonin also significantly increased SL and SVI by 15.7% and 15.6%, with similar or less efficacy than those of 200 μM (Figure 1D,E).

### 2.2. Ultrastructural Alterations in Embryos of Oat Seeds under Aging and Melatonin Priming

**Figure 2:**
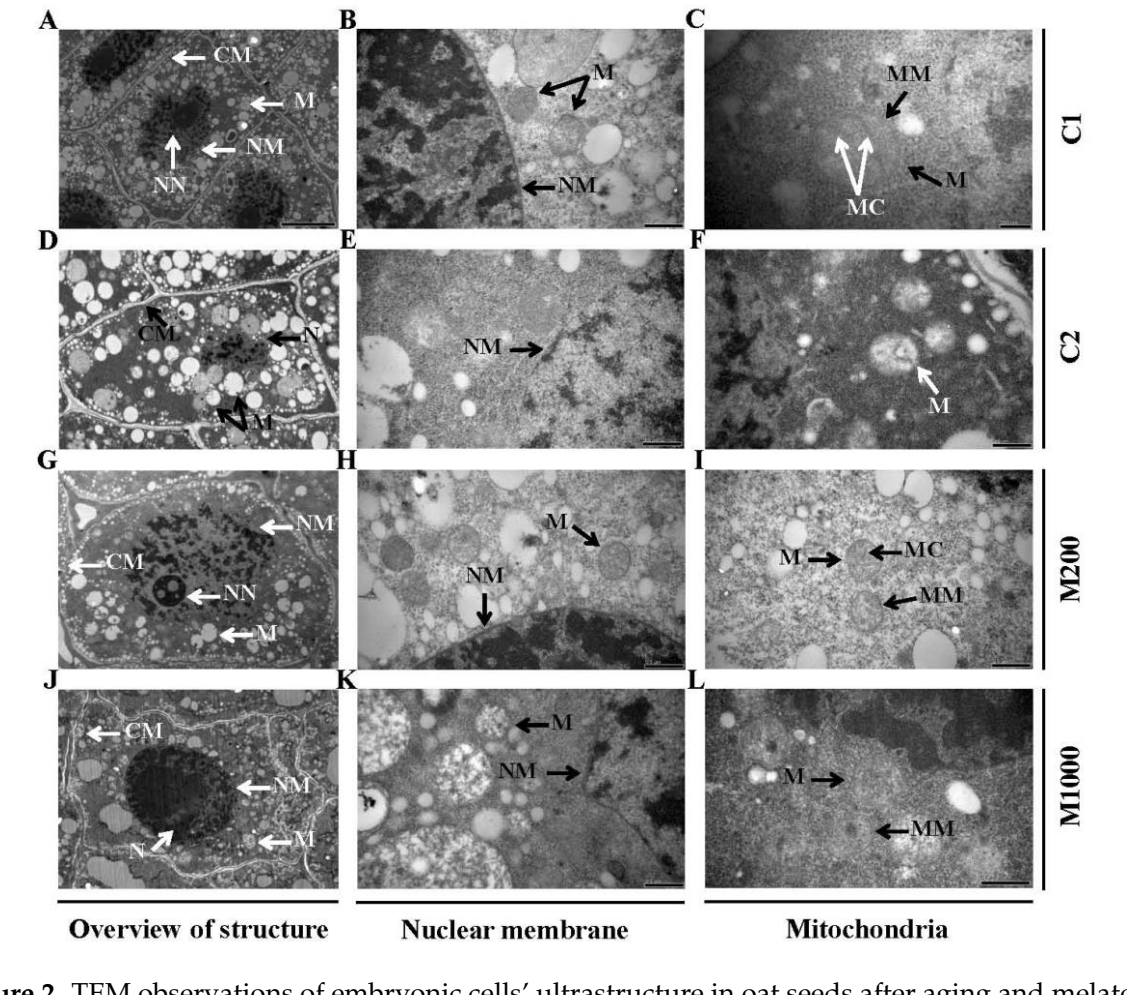
TEM observations of embryonic cells’ ultrastructure in oat seeds after aging and melatonin priming. (**A**–**C**) C1; (**D**–**F**) C2; (**G**–**I**) M200; (**J**–**L**) M1000. (**A**,**D**,**G**,**J**) Overview of embryonic cell’s structure, (**B**,**E**,**H**,**K**) changes of nuclear membrane, (**C**,**F**,**I**,**L**) ultrastructure of mitochondria. CM, cell membrane; N, nuclear; NN, nuclear nucleolus; NM, nuclear membrane; M, mitochondria; MC, mitochondrial cristae; and MM, mitochondrial membrane. Bars = 200 nm (**C**), 0.5 µm (**B**,**E**,**F**,**H**,**I**,**K**,**L**), 2 µm (**D**,**G**,**J**), and 5 µm (**A**).

The TEM images of embryonic cells’ ultrastructure in oat seeds during aging and melatonin priming were acquired (Figure 2). The embryonic cells of non-aged oat seeds (C1) which were imbibed at 20 °C for 12 h exhibited the typical ultrastructure, i.e., intact cytoplasmic membrane and normal distribution of organelles. There was no plasmolysis between membrane and cell wall. In cell nucleus, there were clearly visible nucleolus and complete double-layer nuclear membrane structure. Mitochondria displayed electron-transparent matrix, distinct inner and outer membrane, usually narrow cristae, and typically spherical or ellipsoidal appearance (Figure 2A–C).

Seed aging resulted in damage to embryonic cells, with incomplete cytoplasmic membrane and plasmolysis, and mainly altered the ultrastructure of the cell nucleus and mitochondria. The structural response of the nucleus to aging was that the boundary between the nucleus and cytoplasm became blurred and indistinct with only the location of the nuclear region being vaguely distinguished, nucleolus disappeared, and the double-layer nuclear membrane was destroyed. Mitochondria swelled and burst, with their inner and outer membranes damaged, crista disappeared, and the matrix become thin similar to “vacuole” structure (Figure 2D–F).

Furthermore, the ultrastructure of embryonic cells in melatonin-primed seeds was observed to evaluate whether melatonin could prevent these alterations caused by aging. The results indicated that 200 μM melatonin could restore the integrity of the cellular structure. The boundary between the nucleus and cytoplasm was obvious, and explicit nuclear region, clear inner and outer layers of the nuclear membrane, and visible nucleolus were found. The structure of mitochondria returned to typical, with internal crista and complete double-layer membrane (Figure 2G–I). However, cellular structural integrity could only be partially repaired using 1000 μM MP, as it still showed some slight plasmolysis, partially complete nuclear membrane, and the fuzzy distribution of nucleolus in cell nucleus. This is similar for the mitochondrial ultrastructure, as their membrane bilayer was faintly visible, and the internal crista was still not visible (Figure 2J–L).

In summary, the significant phenotypes in germinability, seedling growth, and the altered ultra-microstructure suggested that various physiological or molecular changes could take place after the processes of seed aging and subsequent melatonin priming.

### 2.3. ROS Generation, Lipid Peroxidation, and Antioxidases’ Changes in Embryos of Oat Seeds under Aging and Melatonin Priming

**Figure 3:**
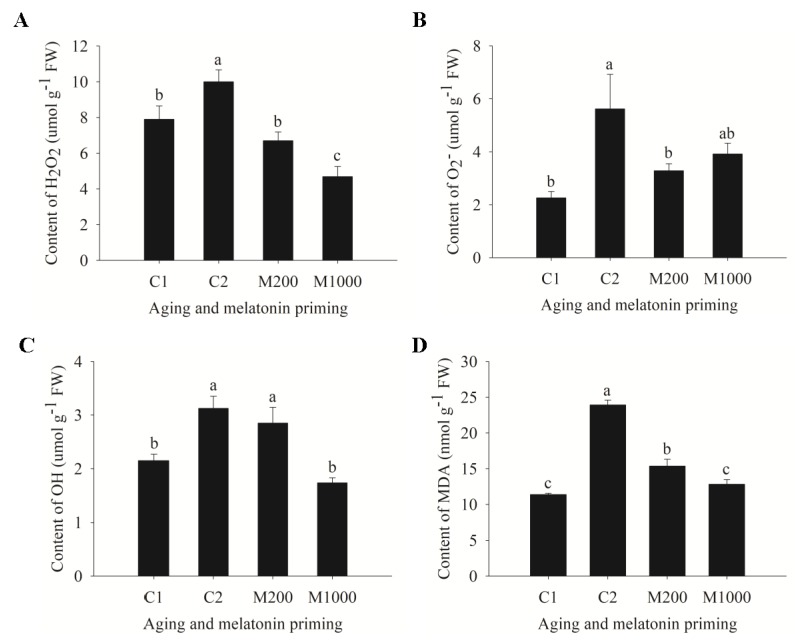
Reactive oxygen species (ROS) accumulation and lipid peroxidation changes in oat’s embryos after aging and melatonin priming. (**A**) H_2_O_2_ content; (**B**) O_2_^−^· content; (**C**) OH· content; (**D**) MDA content. Values were recorded by the means ± SE (*n* = 4). Different letters indicated significant differences of aging and melatonin priming at the *p* < 0.05 level.

Both the ROS (hydrogen peroxide (H_2_O_2_), superoxide anion (O_2_^−^·), and hydroxyl radical (OH·)) production and the malondialdehyde (MDA) accumulation were significantly induced by seed aging, increasing by 26.6%, 148.8%, 45.5%, and 110.1%, respectively (Figure 3). Priming with 200 μM of melatonin (M200) significantly decreased the content of H_2_O_2_, O_2_^−^·, and MDA in aged seeds, by 33.0%, 41.5%, and 35.8%, respectively. However, 1000 μM melatonin (M1000) significantly reduced the content of H_2_O_2_ (by 53.1%), OH· (by 44.5%), and MDA (by 46.6%), which were also significantly lower than those in M200 (Figure 3A,C,D). And there were no significant differences of O_2_^−^· content between M1000 and C2 or M200 (Figure 3B).

**Figure 4:**
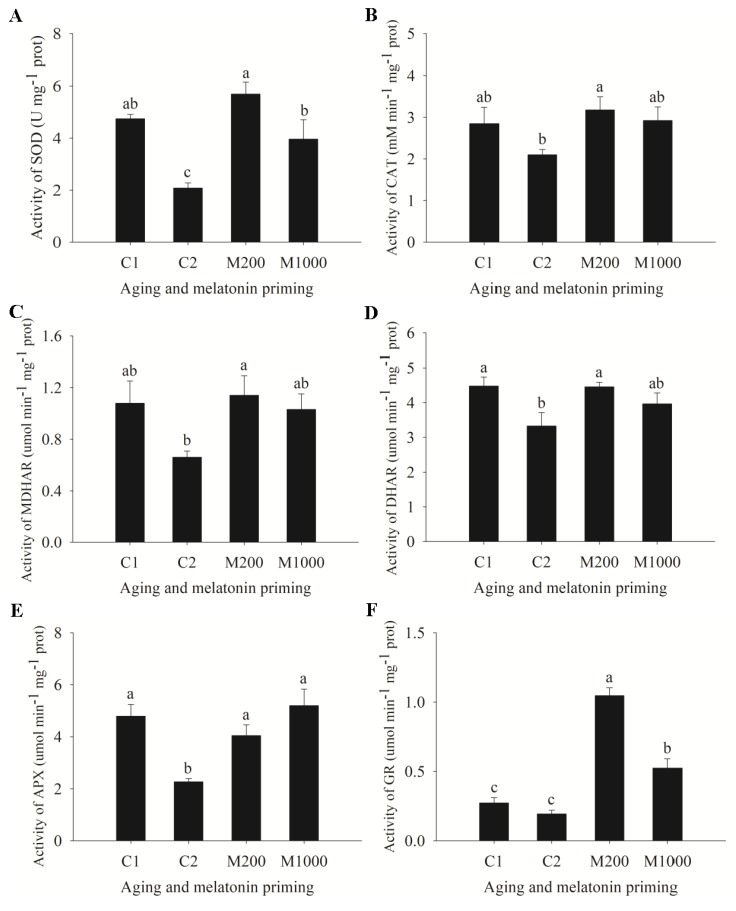
Variation of antioxidant defense function in oat embryos under aging and melatonin priming. (**A**) Activity of superoxide dismutase (SOD); (**B**) Activity of catalase (CAT); (**C**) Activity of monodehydroascorbate reductase (MDHAR); (**D**) Activity of dehydroascorbate reductase (DHAR), (**E**) Activity of ascorbate peroxidase (APX); (**F**) Activity of glutathione reductase (GR). Values were recorded by the means ± SE (*n* = 4). Different letters indicated significant differences of aging and melatonin priming at the *p* < 0.05 level.

In addition, the indices related to antioxidant defense displayed two different trends caused by seed aging, showing that the activities of SOD, dehydroascorbate reductase (DHAR), and APX in C2 were significantly lower than those in C1, and no significant differences were generated in the activities of CAT, monodehydroascorbate reductase (MDHAR), and glutathione reductase (GR) between C1 and C2 (Figure 4). M200 significantly increased the activities of SOD, CAT, MDHAR, DHAR, APX, and GR by 173.8%, 51.2%, 72.5%, 34.0%, 78.5%, and 438.4%, respectively; and there were no significant differences for these enzymes between M200 and C1 except for GR, which was significantly higher than that in C1 (Figure 4). Furthermore, M1000 significantly increased the activities of SOD, APX, and GR, among which SOD and GR activities were significantly lower than those in M200 (Figure 4A,E,F). As for the activities of CAT, MDHAR, and DHAR, there were no significant differences between M1000 and C2 (or M200) (Figure 4B–D).

### 2.4. Proteomics Overview of Embryos in Oat Seeds

To uncover the differential proteins involved in aging and melatonin priming, a global analysis of the iTRAQ-based quantitative proteome was performed, using embryos from non-aged, aged and aged + MP oat seeds. A total of 2643 proteins were identified (Table S1), which were, then, annotated using GO and COG databases. GO annotation showed that the identified proteins were classified into three classes, totalling 52 subsections, including biological process (28), cellular component (11), and molecular function (13), of which the largest group was, respectively, categorized as cellular process, cell and binding with 1991, 2185, and 1443 identified proteins (Figure S2A). Furthermore, the identified proteins were also categorized into 24 clusters of COG categories contained in four groups (Figure S2B).

**Figure 5:**
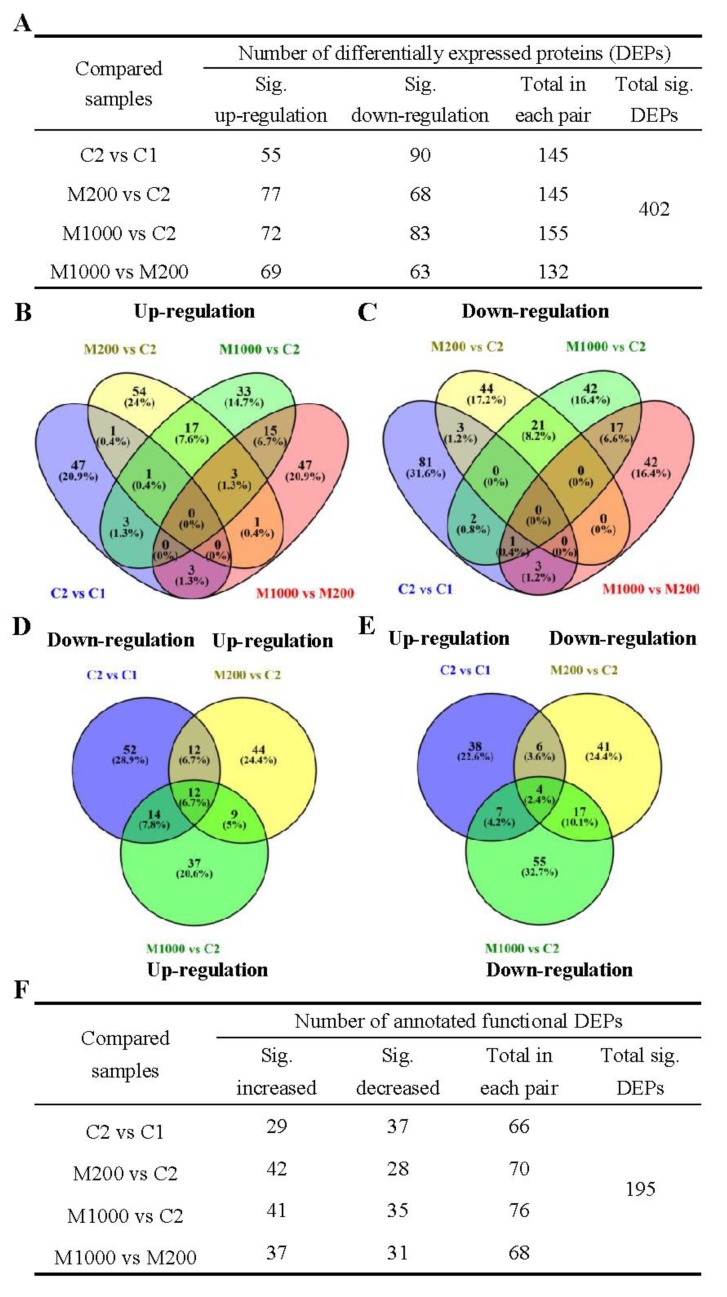
The differentially expressed proteins (DEPs) information identified and quantified in embryo of oat seeds under aging and melatonin priming. (**A**) Number of all DEPs; (**B**) Upregulated DEPs during two processes; (**C**) Downregulated DEPs during two processes; (**D**) The DEPs were downregulated during the aging process, and then upregulated during at least one of the melatonin priming processes (M200 or M1000); (**E**) The DEPs were upregulated during the aging process, and then downregulated during at least one of the melatonin priming processes (M200 or M1000); (**F**) Number of the annotated functional DEPs.

The expression levels of the identified proteins were pairwise compared and analyzed as two groups, i.e., protein alterations during seed aging process (C2 vs. C1) and protein alterations during the melatonin priming processes (M200 vs. C2, M1000 vs. C2, and M1000 vs. M200). Proteins with a fold change (FC) ≥ 1.5 and *p*-value ≤ 0.05 were considered upregulated, whereas those with a FC < 0.67 and *p*-value < 0.05 were downregulated. Taking into account these criteria, among the total 2643 identified proteins, a total of 402 DEPs were identified, among which 145 DEPs that included 55 upregulated and 90 downregulated DEPS were found in aged seeds as compared with non-aged oat seeds (Figure 5A and Table S2). However, during the melatonin priming processes, as compared with aged seeds, 145 DEPs were identified in M200, of which 77 and 68 were upregulated and downregulated respectively; meanwhile, 155 DEPs were identified in M1000, of which 72 and 83 were upregulated and downregulated, respectively (Figure 5A and Table S3).

In total 70 DEPs were shared by the aging process and at least one of the melatonin priming processes (Table S4A). In summary, four different expression profiles were presented among these shared DEPs under two processes, of which the detailed information were listed as follows: Seven DEPs were downregulated under two processes; 39 DEPs were simultaneously identified, downregulated under the aging process, and then upregulated under at least one of the melatonin priming processes (Figure 5D); seven DEPs were upregulated under two processes; 17 DEPs were simultaneously identified, upregulated under the aging process, and then downregulated under at least one of the melatonin priming processes (Figure 5E).

In addition, a total of 225 DEPs were upregulated, of which 47 (20.9%), 54 (24.0%), 33 (14.7%), and 47 (20.9%) DEPs were upregulated only in “C2 vs. C1”, “M200 vs. C2”, “M1000 vs. C2”, and “M1000 vs. M200”, respectively. Meanwhile, one, three, and 17 upregulated DEPs were, respectively, shared by “C2 vs. C1” and “M200 vs. C2”, “C2 vs. C1” and “M1000 vs. C2”, “M200 vs. C2” and “M1000 vs. C2” and one upregulated DEP was shared by “C2 vs. C1”, “M200 vs. C2”, and “M1000 vs. C2” (Figure 5B). Among 256 downregulated DEPs, respectively, 81 (31.6%), 44 (17.2%), 42 (16.4%), and 42 (16.4%) DEPs were only in“C2 vs. C1”, “M200 vs. C2”, “M1000 vs. C2”, and “M1000 vs. M200”; three, two, and 21 DEPs were shared by “C2 vs. C1” and “M200 vs. C2”, “C2 vs. C1” and “M1000 vs. C2”, “M200 vs. C2” and “M1000 vs. C2”, respectively, whereas no DEPs were shared by “C2 vs. C1”, “M200 vs. C2”, and “M1000 vs. C2” (Figure 5C).

Due to the unavailable genome reference for *Avena sativa*, more than half of the DEPs were uncharacterized or predicted proteins, therefore, functional analysis was focused on the 195 DEPs with annotated function (Table S5A). Among the 2643 identified proteins, 66 DEPs were identified during the aging process with 29 upregulated significantly and 37 downregulated as compared with non-aged oat seeds. For the melatonin priming processes, as compared with aged seeds, 70 DEPs were identified in the case of M200, which including 42 upregulated and 28 downregulated DEPs; meanwhile, 76 DEPs were identified in the case of M1000, including 41 upregulated and 35 downregulated DEPs (Figure 5F).

### 2.5. Functional Annotation Analysis of DEPs in Embryos

**Figure 6:**
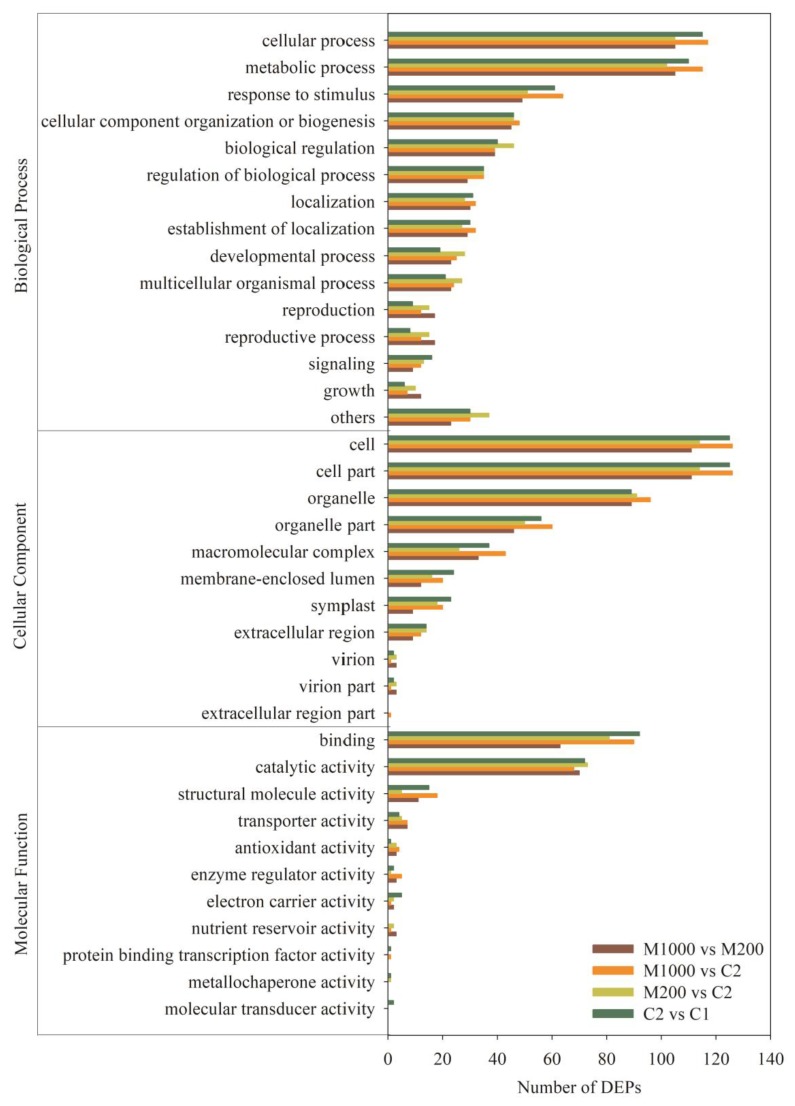
Gene ontology (GO) enrichment analysis of all DEPs in the embryo of oat seeds under aging and melatonin priming processes. The following three changes were analyzed: the DEP changes during seed aging process (without melatonin priming, C2 vs. C1); the effect of melatonin priming on DEPs (M200 vs. C2 and M1000 vs. C2); and the difference of various melatonin concentrations (M1000 vs. M200). Note: “Others” in “Biological Process” contained pigmentation, nitrogen utilization, viral reproduction, rhythmic process, cell proliferation, death, immune system process, negative regulation of biological process, positive regulation of biological process, and multi-organism process.

To understand the DEPs during aging and the melatonin priming processes, all DEPs were submitted to the Uniprot database for analysis of functional annotation. The GO enrichment analysis, to illuminate functional distribution of DEPs during two processes, demonstrated that DEPs were classified into biological process, cellular component, and molecular function, including 24, 11, and 11 categories, respectively (Figure 6). For biological process, the DEPs were mainly involved in the cellular process and metabolic process. In the cellular component category, the DEPs were mainly enriched in the cell, cell part, and organelle. The most abundant DEPs in molecular function category were related to binding and catalytic activity. These results showed that the majority of DEPs were involved in the cellular process, metabolic process, binding and catalytic activity, suggesting that aging and melatonin priming mainly affected the physiological and cellular metabolic events in oat seeds.

**Figure 7:**
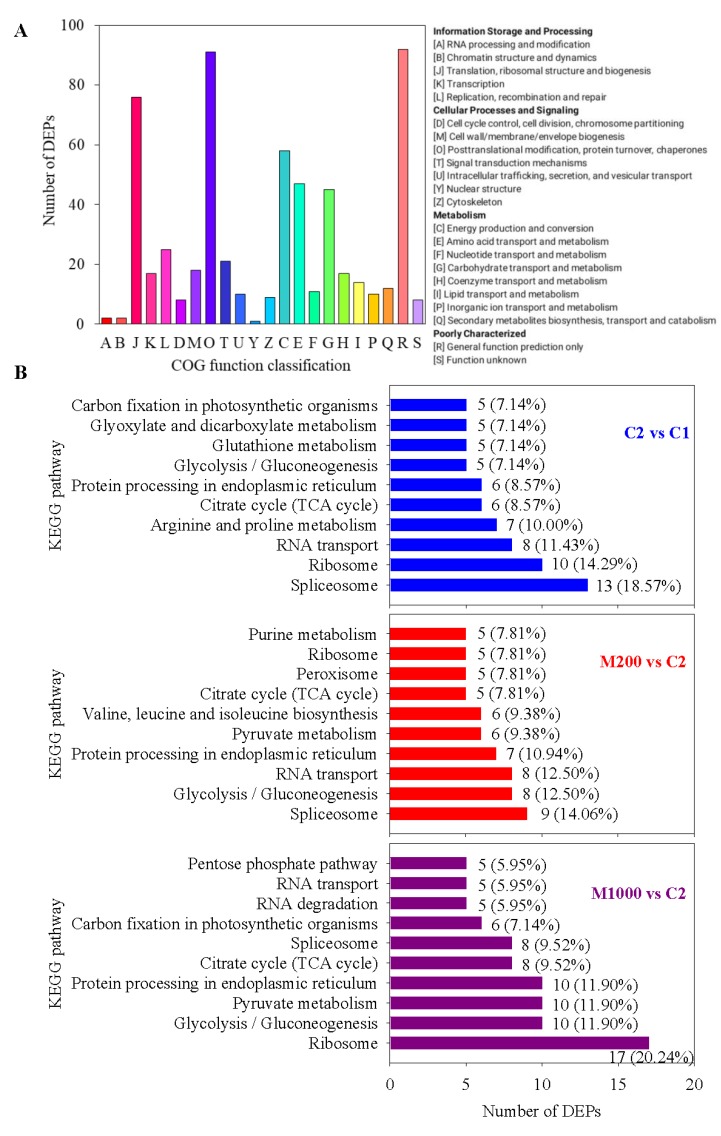
Function classification of all DEPs in embryo of oat seeds under aging and melatonin priming. (**A**) Cluster of orthologous groups (COG) function annotation; (**B**) Top 10 of Kyoto Encyclopedia of Genes and Genomes (KEGG) function classification.

Of the 402 DEPs, 318 were categorized into four groups including 22 clusters, based on the COG database (Figure 7A). These DEPs were mainly involved in translation, ribosomal structure, and biogenesis (12.8%); posttranslational modification, protein turnover, chaperones (15.3%); energy production and conversion (9.8%); amino acid transport and metabolism (7.9%); and carbohydrate transport and metabolism (7.6%). Detailed annotation information of DEPs categorized into different clusters is listed in Tables S5B,C.

To further investigate the functions of the DEPs during aging and melatonin priming processes, a Kyoto Encyclopedia of Genes and Genomes (KEGG) pathway analysis was also performed. According to the Top 10 KEGG pathways (Table S6), the results indicated that the aging, as well as the M200 and M1000 treatments affected spliceosome (18.57%, 14.06%, and 9.52%), ribosome (14.29%, 7.81%, and 20.24%), RNA transport (11.43%, 12.50%, and 5.95%), citrate cycle (TCA cycle) (8.57%, 7.81%, and 9.52%), protein processing in endoplasmic reticulum (8.57%, 10.94%, and 11.90%), and glycolysis/gluconeogenesis (7.14%, 12.50%, and 11.90%). In addition, KEGG terms including arginine and proline metabolism and glutathione metabolism were highly enriched in aging, whereas other KEGG terms containing pyruvate metabolism, valine, leucine and isoleucine biosynthesis, peroxisome and purine metabolism were considerably enriched in M200, and KEGG terms comprising pyruvate metabolism and RNA degradation were greatly enriched in DEPs in M1000 (Figure 7B).

### 2.6. Validation of Transcriptional Expression Analysis of Selected DEPs by qRT-PCR

To validate the proteomic data, avoiding the possible error of a single test method, qRT-PCR was performed to further verify the correspondence between protein abundance and their expression at mRNA transcriptional level (Table 1). A total 13 top-expressed DEPs were selected, including four, five, five, and six DEPs in “C2 vs. C1”, “M200 vs. C2”, “M1000 vs. C2”, and “M1000 vs. M200”, respectively, to quantify their transcript level of mRNA. The transcript level of six genes (W5DQ12, A0A1Y1HNC9, A0A1D5RZK8, Q10P35, W5AA91, and A0A1D5VEP2) showed the consistencies with their corresponding proteins.

## 3. Discussion

Seed aging during storage is irreversible and results into the decrease or even loss of seed vigor, which in turn leads to serious agricultural problems [5]. Delayed germination and subsequent slow growth are two major characteristics of aged seeds [9]. It has been reported that seed germination or seedling growth is suppressed in aged seeds, such as rice [32] and oat [33]. In this study, oat seed vigor was significantly reduced as the GP decreased from 100% to 70% after 48 d aging (Figure 1A), together with decreased GI and VI (Figure 1B), and depressed 10-days seedling growth (Figure 1C–E). In addition, the ultrastructural alterations were accompanied in the embryonic cells of oat seeds (Figure 2D–F). Exogenous melatonin promotes seed germination or seedling growth under various stress conditions [34]. For instance, melatonin has been demonstrated to promote seed germination under high salinity in cucumber [35], and significantly improve coleoptile length, seedling fresh weight, and dry weight in wheat under osmotic stress [36]. In this study, the results indicated that 200 μM melatonin significantly retarded the damage caused by aging to germination and seedling growth, improving GP, VI, GI, SL, SW, and SVI (Figure 1), as reported, to increase the activity of aged seeds and enhance the growth of germ and radicle in maize [27]. It has also been demonstrated that melatonin had an ameliorative effect on meristematic cells in *Vigna radiata* roots under chilled and rewarmed conditions [37]. Similarly, our results also showed that melatonin could repair structural damage of radicle cells caused by aging, integrating the cell membrane, nucleus, and mitochondria, and the 200 μM priming worked better (Figure 2G–I). This finding was consistent with the germination results, which revealed that the structural restoration of cells and highly active organelles were highly correlated with promoting the germination and seedling growth in aged oat seeds.

Seed embryo is an important tissue where seed physiological aging and germination occur in oat. Here, the iTRAQ-based quantitative proteomics was adopted in order to study the embryonal protein changes during the process of seed aging and melatonin priming. On the basis of the proteomic data, a total of 402 DEPs were identified in the two processes and these DEPs were found to be related to multiple pathways, mainly including carbohydrate and energy metabolism (sucrose metabolism, glycolysis, pyruvate metabolism, TCA cycle, and fatty acid metabolism), protein synthesis (ribosome), and amino acid metabolism.

Seed aging is a naturally occurring event during storage, and priming treatment could promote germination and improve seedling consistency. When subjected to priming, seed embryonal cells transform from a static state into a highly active metabolic state, in which various physiological and cellular processes are initiated, including energy supply, protein synthesis, amino acid metabolism, and stress-related activities [11]. According to the proteomics analysis, it could be illustrated that seed aging and melatonin priming induced plentiful DEPs in embryo of oat seeds. According to the results of annotated functional proteins, more downregulated DEPs were detected in aged embryos relative to non-aged embryo, whereas more upregulated DEPs were observed in melatonin-primed embryos versus aged embryos (Figure 5F). However, more interestingly, melatonin priming reversed the expression patterns of certain DEPs in the above two comparisons (Table S4B), implying that they could be involved in melatonin-regulated expression under aging condition. Many identified DEPs in this study would be helpful to reveal the underlying mechanism of seed aging and provide a theoretical basis for optimizing the technology of promoting seed germination.

Many DEPs were significantly enriched in sucrose metabolism, glycolysis, TCA cycle, and fatty acid metabolism. Sucrose synthase (SuSy) and sucrose-phosphate synthase (SPS) are two key enzymes in plant sucrose metabolism [38], respectively, catalyzing the conversion of sucrose into UDP-glucose (UDPG) and fructose, and the production of sucrose-6-phosphate (S6P) as the central enzyme in sucrose synthesis. It had been reported that SuSy was a downregulated protein in the aging of coix (*Coix lacryma-jobi* L.) seed during storage [39]. In agreement with our findings, the expression of SuSy (A0A0D3FJJ4) and SPS2 (Q6EZE7) was downregulated in embryos of aged oat seeds (Table S5B), indicating that aging reduced sucrose hydrolysis.

Glycolysis, a metabolic pathway for energy production that converts glucose into pyruvate, which is, then, converted to acetyl-CoA, plays an important role in plants [40]. Proteins related to energy supply have significant effects on seed vigor, and in wheat, glycolysis has been reported to provide energy for seedling formation, development and growth in seed germination [41]. In our study, the abundance of phosphoglycerate kinase (PGK, A0A1D6AGT9), involved in one of two substrate phosphorylation reactions during glycolysis via transferring the phosphate group from 1,3-bisphosphoglycerate (BPGA) to form 3-phosphoglycerate (3-PGA), decreased under aging condition in oat seeds (Table S5B). In addition, phosphoenolpyruvate carboxylase 2 (PEPCase2, P29194), an enzyme in the carboxyl lyase family that could carboxylate phosphoenolpyruvate (PEP) to oxaloacetic acid (OAA) in the cytoplasm [42], was also decreased by aging (Table S5B). Mira et al. [43] found that, in lettuce (*Lactuca sativa* L.) seeds, there was a strong correlation between glycolysis related byproducts and aging. Therefore, our findings suggested that the disordered energy metabolism caused by PGK and PEPCase2 could result in seed aging in oat. Whereas, the data also showed that 200 μM melatonin priming significantly upregulated the expressions of PEPCase2, enolase 2 (ENO2, Q10P35), and pyruvate kinase (PK, Q8S7N6) by approximately 5.32-, 7.78- and 15.62-folds, respectively as compared with aged oat seeds (Table S5C). Enolase, a key enzyme that catalyzes the conversion of 2-phosphoglycerate (2-PGA) into phosphoenolpyruvate (PEP) [44], and PK that further catalyzes the last step to form pyruvate, play critical roles in glycolysis. Cui et al. [36] reported, in wheat seedlings, that melatonin enhanced ENO level in response to osmotic stress. The KEGG function classification also supported a role of melatonin in regulating glycolysis (Figure 7B). These results indicated that the glycolytic-related DEPs and the melatonin-regulated expression could be directly associated with germination and seedling growth in aged oat seeds.

As the end-product of glycolysis, pyruvate could be further converted into acetyl-CoA through the pyruvate dehydrogenase complex (PDHC), including three key enzymes of pyruvate dehydrogenase, dihydrolipoyl transacetylase, and dihydrolipoyl dehydrogenase (DLD) [45]. Acetyl-CoA serves as an important intermediary for biosynthesis and the TCA cycle in plants. In this study, it was found that the DLD level was significantly higher in aged seeds than in non-aged seeds, and moreover, 200 μM melatonin significantly increased the expression of pyruvate dehydrogenase E1 component subunit alpha (PDHA, A0A0D3FS26) and pyruvate dehydrogenase E1 component subunit beta (PDHB, A0A1J7GPG1, F2DDZ5) (Table S5C). These results suggested that acetyl-CoA could be increased from pyruvate through a positive intracellular conversion to meet self-sufficiency need or melatonin induced passive conversion, providing more reaction substrates for the TCA cycle. The TCA cycle is a key metabolic pathway to provide energy for cells, which unifies the metabolism of carbohydrate, fat, and protein [46]. As mitochondrial proteins, aconitate hydratase (ACO) catalyzes the conversion of citrate into isocitrate and malate dehydrogenase (MDH) catalyzes malate into oxaloacetate. Xu et al. [39] found that the ACO expression in coix seed increased after 5 months of storage at room temperature which indicated that seed respiration was enhanced, and energy consumption was increased. Moreover, in soybean seeds, preharvest seed deterioration led to a significant high level of MDH under high temperature and humidity stress [42]. Consistent with these studies, ACO (K3YG24) and MDH (V4KRX5) levels were significantly higher in aged oat seeds (Table S5B). However, another key enzyme in the TCA cycle, citrate synthase (CS, A0A1D5YZH3), was significantly inhibited by aging. Therefore, the upregulation of ACO and MDH and the downregulation of CS could imply that the mitochondria in oat seeds after 48-days aging still maintained the certain ability and the stability of normal function and attempted to provide enough energy for cell metabolism by enhancing TCA, and therefore resist the damage caused by aging. In addition, DEP related to energy production such as ATP synthase subunit beta (A0A1D5SCJ1) was also detected and increased in response to melatonin priming (200 μM).

Lipid is metabolized via β-oxidation to form acetyl-CoA, which enters into the TCA cycle and is eventually converted to hexose for seedling growth after germination [47]. It was found that the germination and post-germination growth of *Arabidopsis* mutants with β-oxidation destruction was impaired, when they germinated on medium without sucrose [48]. 3-Ketoacyl-CoA thiolase (KAT) catalyzes the fourth step of the β-oxidation degradation pathway, through converting 3-ketoacyl-CoA to acetyl-CoA. Here, one 3-ketoacyl-CoA thiolase-like protein (KATLP, D2KZ12) involved in lipid degradation was downregulated 0.57-fold by seeds aging (Table S5B). After melatonin priming, KATLP and peroxisomal fatty acid beta-oxidation multifunctional protein (MFP, B6SXV4) were remarkably upregulated by 1.71- and 2.48-folds, respectively. These results suggested that β-oxidation in oat was impaired, and therefore failed to provide sufficient energy for germination and seedling growth. However, melatonin rescued the malignant consequence, indicating that β-oxidation of lipid could be the main energy producing way in primed seeds, and the provided energy was conducive to maintaining the stability of TCA’s normal function which, in turn, supplied stable substrates and energy for protein synthesis and other metabolic events. Furthermore, during the seed aging, the integrity of membrane structure decreases due to the enhancement of membrane lipid catabolism, and lipid-degrading enzymes such as phospholipase D (PLD) seems to be involved in this process [49]. In this study, the expression of PLD (A9UIF0) was downregulated by 200 μM melatonin, which indicated that melatonin protected the integrity of the cell membrane via reducing the catabolism of membrane lipids. This result was also consistent with the structural repair of the cell membrane by TEM observation (Figure 2).

Proline is deemed to clean ROS and reduce the damage caused by abiotic stress in plants. Under adverse conditions, cells can control proline content by increasing proline synthase expression or inhibiting proline degradation. Chen et al. [50] found that the proline content in aged oat seeds decreased significantly and, consequently, seed vigor decreased. Furthermore, δ-1-pyrroline-5-carboxylate synthetase (P5CS) catalyzed the first step of proline synthesis, and the overexpression of *P5CS1* improved drought tolerance in rice [51]. The data showed that the P5CS (Q53UC8) expression was downregulated by 0.31-fold in embryos (Table S5B), indicating that the inhibition of proline synthesis caused by aging could be another reason of seed vigor and germination declining.

Aspartate aminotransferase (AspAT) catalyzes the reversible transamination between aspartate and 2-oxalate to produce glutamate and oxaloacetate, which plays a key role in the distribution of carbon and nitrogen in plants [52]. Glutamate decarboxylase (GAD) is a pyridoxal phosphate (PLP)-dependent enzyme that catalyzes the irreversible α-decarboxylation of L-glutamate to produce γ-aminobutyric acid (GABA) [53]. A previous study proved that GABA, under the action of GABA transaminase, could transaminize with pyruvate to produce succinate semialdehyde (SSA), which was, then, oxidized to form succinate and entered into the TCA cycle [54]. In this study, seed aging resulted in the downregulated expression of AspAT (F2DCC0) and GAD (A0A1D5VZC0) in oat (Table S5B), which suggested that the reduced supply amino acids involved in aspartate and glutamate metabolism could limit the germination and seedling growth of oat seeds.

Moreover, after aging of oat seeds, the expressions of D-3-phosphoglycerate dehydrogenase (PHGDH, A0A1D5URU6) and serine hydroxymethyltransferase (SHMT, A0A0D3HLJ4) were upregulated by 2.31- and 2.09-folds, respectively, which played key roles in serine biosynthesis (Table S5B). Seven *SHMT* genes were found in *Arabidopsis* [55], and *SHM1* was reported to play a key role in regulating cell damage caused by abiotic stress. Additionally, the level of methionine synthetase 1 enzyme (MS1, Q4LB13), involved in methionine metabolism, was increased by 2.27-fold after aging (Table S5B), and this result was consistent with the change of MS1 in rice seed embryos after artificial aging (100% relative humidity, 40 °C) [5]. The sulfur-containing amino acids and their metabolism play a pivotal role in determining whether seeds could succeed to germinate completely [56], and our findings indicated that the damage of seed aging could be resisted by enhancing methionine synthesis related enzymes. Except as the structural unit for protein synthesis, methionine also participates in the synthesis of S-adenosylmethionine (SAM), catalyzed by the fundamental enzyme S-adenosylmethionine synthase (SAMS). The SAM is a major methyl donor in plant metabolism, which is involved in the transmethylation of many secondary metabolites and serves as a synthetic precursor of polyamines (Put, Spd and Spm) and ethylene [57]. But the SAMS (K3XHU5) in aged oat seeds was downregulated (Table S5B), suggesting that aging inhibited the formation of methyl donor SAM, which could confirm the proposition of Catusse et al. [58] that the active methyl cycle played a crucial part in seed vigor. Priming with 200 μM melatonin upregulated the abundance of SHMT (A0A1D5SFH5 and A0A1J3K1Q3) and phospho-2-dehydro-3-deoxyheptonate aldolase (PDDA) by 1.99- to 2.99- and 1.54-folds, respectively. The PDDA, an enzyme in the upstream biosynthesis pathway of amino acids, was involved in the biosynthesis of phenylalanine, tyrosine, and tryptophan. Light and Anderson [59] reported that PDDA-1 was located at the key regulatory point for the synthesis of the above three aromatic amino acids, and further involved in the biosynthesis of chorismic acid, an important intermediate that led to synthesis of various basic metabolites and lignin.

The deleterious effects of aging on seed germination are related to the protein synthesis system. As the organelles catalyzing protein synthesis, ribosomes consist of a large subunit and a small subunit [60]. In this study, the expression of 50S ribosomal protein L14 (RPL14, B6T366), 60S ribosomal protein L36 (RPL36, A0A1E5V7S7), 40S ribosomal protein S2 (RPS2, B6TNR8), and 40S ribosomal protein S27 (RPS27, A0A1Q3DHJ5) were downregulated under aging condition, while 60S ribosomal protein L12 (RPL12, B4FRM7) and 60S ribosomal protein L30 (RPL30, B6U9H6) were upregulated (Table S5B). Hence, the expression levels of most ribosomal subunits were decreased during the aging. Herein, the protein level of one ribosomal small subunit family protein (RPS27) was induced by 200 μM melatonin under aging stress (Table S5C), which manifested that the ribosome pathway took part in the melatonin-mediated aging stress responses. Eukaryotic translation initiation factor 3 (eIF3) plays an important role in protein translation, and Xu et al. [39] found that eIF3 was downregulated in coix seeds after storage at room temperature for 10 months. Rajjou et al. [61] also reported that the downregulation of translation related proteins during seed aging resulted in the delay of protein synthesis in germination. The decrease of eIF-3E (A0A0D3GNQ3) and eIF-3H (B4FR57) in embryos (Table S4B) meant that oat seeds became weak on the protein translation after aging. While melatonin priming significantly increased the levels of eIF-3E, eIF-3H, and 60S ribosomal export protein NMD3 (F2DGV9). NMD3 is an adapter that exports 60S ribosome subunits from the nucleus, which binds to the new 60S subunits and recruits the export receptor Crm1 to facilitate passage through the nuclear pore complex [62]. In brief, these results showed that melatonin improved protein translation in aged oat seeds.

The folding of new polypeptides into mature proteins is regulated by chaperonins, heat shock proteins, and other protein processing related catalysts. Accordingly, the significantly increasing chaperonin (Q10RW9) and significantly decreasing heat shock protein 83 (HSP83, A0A0A7LU49), involved in endoplasmic reticulum protein processing, suggested that aging affected the protein processing, and then further could lead to protein damage. Nevertheless, melatonin increased the expression of 70 kDa heat shock cognate protein 1 (HSC-I), another molecular chaperone that helps protect cells against multiple stresses by repairing damaged proteins, indicating that the protein folding, denaturation, and degradation were improved in melatonin primed seeds. In addition, several identified DEPs related to proteolysis were also detected in this study. The ubiquitin-proteasome pathway is in charge of the elimination of proteins induced by misfolding, destruction, potential toxicity, and various cellular stresses in all phases of normal plant developmental events [63]. As an important protein degradation pathway, it was found to regulate protein turnover during seed germination when exposed to smoke-water and the active compound [64]. Sekar et al. [65] reported that 26S proteasome subunit in black gram (*Vigna mungo* L.) seeds was upregulated during artificial aging, indicating that aging promoted the production of damaged proteins and the substantial destruction of storage proteins, therefore, higher active 26S proteasome was required to perform the cleanup task. During aging of oat seeds, proteasome subunit beta type (PSB, W5DQ12) was identified as a downregulated protein, and melatonin altered the expression of the other proteasome subunit alpha type (PSA, A0A1J3K4N2) (Table S5C). The PSA participates in the recognition and degradation of ubiquitinated proteins and plays an important role in plant defense through protein degradation [66]. Taken together, these findings demonstrated that aging led to the reduced eliminating capacity of proteasome for protein misfolding, destruction, and melatonin changed the improper action, which could be the cause of the promoted germination of aged oat seeds.

Calcium signaling is an important regulator in response to stress in many plants [67]. Calcium-dependent protein kinase (CDPK), as the second messenger, can be activated by trace free Ca^2+^ and has a role in the signal transduction pathway. CDPK is responsible for protein phosphorylation by transferring ATP phosphate to protein substrates, therefore, protein phosphorylation plays an essential role in many signaling pathways, such as cold, heat shock, salt, and ABA stress [68]. In this study, the expression of CDPK19 (P53683) was downregulated in aged oat seeds (Table S5B), suggesting that calcium signaling could be related to oxidative stress induced by aging. Meanwhile, PTI1-like tyrosine-protein kinase 3 (B6TJX6) was also decreased by aging, one protein participated in plant-pathogen interaction pathway and played an important role in signal transduction mechanism.

Various antioxidant mechanisms have evolved in plants to adapt to stress, such as ROS scavenging enzymes and proteins. In this study, several DEPs were identified, including thioredoxin (TRX), glutaredoxin (GRX), peroxiredoxin (PRX), and glutathione S-transferase (GST), which participated in the GPX pathway and the PRX/TRX pathway. TRX, a small conservative protein involved in plant oxidative stress response, operated as the antioxidant to restrict stress through scavenging hydrogen peroxide and certain radicals directly or served as a reductant for the regulation of several ROS-related enzymes, such as CAT, GR, GPX, and PRX [69]. Similar to TRX, GRX can reduce peroxiredoxin acting as a dithiol reductant and GSH-dependent oxidoreductase [70]. Here, aging upregulated one TRX (Q7FT21) and one glutaredoxin homolog 1 (GRXH1, B6THA1); when primed with 200 μM melatonin, TRX was upregulated while GRXH1 was downregulated (Table S5C). In addition, PRX (A0A1Y1HP96) and GST (Q9SP56) were increased by melatonin at the protein level (Table S5C). PRX is a thiol-based peroxide reductase involved in redox status regulation that plays a defensive role by reducing peroxides, peroxynitrites, and excessive hydrogen peroxide in plants [71]. GST is deemed to catalyze the conjugation of GSH to cytotoxic substrates, as a detoxification enzyme, and appears to be necessary for seed germination. Thus, the redox related proteins identified in this study by proteomic analysis could play important roles in melatonin-regulated germination of aged oat seeds. Overall, these results proposed that the upregulation of TRX, PRX, and GST by melatonin, together with the improved antioxidant capacity in AsA-GSH cycle (Figure 4), could, in turn, enhance the ROS detoxification and oxidative stress tolerance of aged oat seeds.

## 4. Materials and Methods

### 4.1. Seed Material

Oat (cultivar “Cayuse”) seeds were available from Rytway Ecotechnology Company (Beijing, China), with the original GP and the moisture content (MC), respectively, being 100% and 8.9% (fresh weight basis). Upon reception, seeds with uniform sizes and weights were selected through a 2.5 mm sieve, dehulled (removing the palea and lemma) and adjusted to 10% MC according to the saturated salt solution equilibrium static weighing method. Then, seeds were immediately sealed in hermetical aluminum foil bags (120 × 170 mm, approx. 25 g in each bag) and stored at −20 °C in the dark prior to further experimentation.

### 4.2. Experimental Treatments

Oat seeds of 10% MC in bags (approx. 25 g each) were incubated in an electric thermostatic cistern (CU-600, China) at 45 °C for 48 d to prepare the aged seeds of 70% GP (determined on the basis of a reverse ”S-shaped” aging curve, Figure S1A), which were used for melatonin priming treatments.

A single layer of aged seeds was placed into a petri dish (110 × 110 mm) with a single layer of filter paper (Guangda Company, China), to which the embryos were tightly attached. After that, aged seeds were primed with 1, 10, 100, 200, 500, and 1000 μM of melatonin solutions (15 mL, immersing embryos) at 20 °C for 24 h in darkness. The ratio of seed weight to melatonin solution volume (*w/v*) was 1:3, and melatonin solutions were changed at 12-hour intervals. After the scheduled priming treatments, seeds were washed in distilled water three times, surface-dried with filter paper, and air-dried back to 10% MC at 20 °C and 33% relative humid atmosphere. Three groups were prepared: (a) normal control, non-aged and non-primed seeds (C1); (b) aging control, aged but non-primed seeds (C2); and (c) melatonin treatments, aged seeds primed with various concentrations of melatonin (marked as M1, M10, M100, M200, M500, and M1000, respectively). After melatonin priming treatments, the dried seeds were used to determine germinability, including GP, VI, and GI.

On the basis of the preliminary results of germinability (Figure 1A,B), the concentrations of 200 μM and 1000 μM were selected for further experiments, including seedling growth, and ultrastructural, physiological, and proteomic analyses. The seeds, with embryos tightly attached to filter paper in a petri dish, were imbibed with 15 mL of distilled water for 12 h at 20 °C in darkness. After imbibition, embryos with the radicle just protruding through the seed coat (Figure S1B), were collected by the isolation of seeds on ice with a scalpel, immediately frozen in liquid nitrogen, and then stored at −80 °C until physiological analysis, protein extraction, and qRT-PCR assays. For ultrastructural observation, isolated embryos were fixed in glutaraldehyde solution.

### 4.3. Germination Test and Seedling Growth Assay

The germination test was carried out according to the criterion of ISTA Rules chapter V [72]. In short, four replicates of 50 seeds each were germinated in petri dishes, with three layers of filter paper moistened with 10 mL of distilled water. Afterward, seeds were incubated in a germination incubator (GXZ-380B, China) at a constant temperature set to 20 °C, with a photoperiod of 8 h light and 16 h dark. Normal seedlings without lesions or defects in morphology were applied as a criterion to assess seed germination, and the number was recorded daily over a period of 10 d. At the end of the 10th day, all normal seedlings of each replicate were taken out, and SL (measured from the embryo to the tip of the longest leaf), SW (fresh basis), and SVI were assayed [73]. The GP, VI, and GI were calculated.

### 4.4. Ultrastructure by Transmission Electron Microscopy (TEM)

The dissected embryos were randomly selected, and radicle tissues were cut into cross sections and fixed into 4% glutaraldehyde solution for 48 h, before being stored at 4 °C condition. The other steps to prepare samples for TEM were referred to the method of Yan et al. [74].

### 4.5. Determination of Physiological Parameters

MDA content was determined to evaluate the LPO according to methods from Bailly et al. [75]. Embryo samples (0.2 g) were ground into powder with liquid nitrogen, homogenized in 4 mL of 5% (*w/v*) trichloroacetic acid, and then centrifuged at 15,000× *g* for 20 min at 4 °C. Reaction mixture contained 2.5 mL of 0.5% thiobarbituric acid in 5% (*w/v*) trichloroacetic acid and 2.5 mL of supernatant. The mixture was incubated at 100 °C for 15 min, immediately cooled to 25 °C and centrifuged at 4000× *g* for 20 min. The MDA content was determined by measuring absorbance at 532 nm and 600 nm.

The ROS including H_2_O_2_, O_2_^−^·, and OH· were determined, respectively, using H_2_O_2_, O_2_^−^·, and OH· Assay Kit, according to the manufacturer’s instructions.

For extraction of antioxidant enzymes, embryo samples (0.2 g) were ground into powder with liquid nitrogen, homogenized in 4 mL of phosphate buffer (50 mM, pH 7.0, containing 1.0 mM EDTA, 1% PVP), and then centrifuged at 15,000× *g* for 20 min at 4 °C. As for APX activity assay, 0.5 mM AsA was added into the phosphate buffer. The resulting supernatant was used for assays of antioxidant enzyme activities.

SOD activity was assayed on the basis of its ability to inhibit the photochemical reduction of nitroblue tetrazolium (NBT) [76]. One unit of SOD activity was defined as the suppression of 50% NBT photochemical reduction at 560 nm. CAT activity was measured by the dynamic change in absorbance at 240 nm in one minute due to the decline of H_2_O_2_ extinction [77]. Supernatant was mixed with 1 mL of phosphate buffer (25 mM, pH 7.0, containing 0.1 mM EDTA) and 0.2 mL of 10 mM H_2_O_2_. MDHAR was determined by measuring the decrease in absorbance at 340 nm as a result of NADH oxidation [78]. Reaction mixture contained phosphate buffer (50 mM, pH 7.0), 1 mM AsA, 0.2 mM NADH, 0.2 U ascorbic acid oxidase, and enzyme extract. DHAR was assayed by monitoring the increase in absorbance at 265 nm because of ascorbic acid formation [79]. Reaction mixture contained phosphate buffer (50 mM, pH 6.3), 1 mM DHA, 10 mM GSH, and enzyme extract. APX activity was measured by monitoring the decrease in absorbance at 290 nm over one minute by reason of ascorbic acid oxidation [80]. Reaction mixture consisted of phosphate buffer (50 mM, pH 7.0), 0.1 mM H_2_O_2_, 0.5 mM AsA, and enzyme extract. GR activity was assayed by measuring the decrease in absorbance at 340 nm due to NADPH oxidation [81]. Reaction mixture contained phosphate buffer (50 mM, pH 7.8), 5 mM MgCl_2_, 0.5 mM GSSG, 1.5 mM NADPH, and enzyme extract. The protein content was quantified referring to the protein quantitative kit. All assays were repeated four times.

### 4.6. Embryonic Protein Extraction and Quantification

Already prepared embryo samples (stored at −80 °C) were used for protein extraction, with two biological replicates (C1, C2, M200, and M1000). The embryos (~0.2 g) were ground into powder in liquid nitrogen, extracted with 200 μL of cold lysis buffer (50 mM Tris-HCl, pH 8.0, 8 M Urea, 2 M Thiourea, 0.1% SDS), suspended using an ultrasonic processor (SCIENTZ-JY92-11N, Ningbo, China) for 15 min, and then centrifuged at 13,000× rpm for 20 min at 4 °C. The supernatant was mixed well with 800 μL of cold acetone (containing 10 mM DTT), and incubated for ~2 h before being centrifuged at 13,000× rpm for 20 min at 4 °C. The collected precipitate was resuspended with 800 μL of cold acetone (containing 10 mM DTT), and then centrifuged at 13,000× rpm for 20 min at 4 °C. The pellet was air-dried and resuspended with 100 μL of lysis buffer. Protein concentration was quantified, and then stored at −80 °C for the next use.

### 4.7. Protein Reduction, Digestion, and iTRAQ Labeling

Proteins (100 µg) were mixed with 10 mM DTT and incubated at 37 °C for one hour, and then 55 mM iodoacetamide was added to alkylate at room temperature for one hour in the dark. After reduction and alkylation, 2 μg of trypsin (Promega, USA) was added at a protein/trypsin ratio of 50:1, and then digested at 37 °C for 12 h, followed by centrifugation at 12,000× rpm for 15 min. After trypsin digestion, acidulation was conducted by adding an equal volume of 0.1% formic acid, then purified by a Strata-X C18 column (Phenomenex, 8B-S100-UBJ) for three times, washed twice with 0.1% formic acid + 5% acetonitrile, and eluted with 0.1% formic acid + 80% acetonitrile. Peptides were evaporated to dryness by vacuum centrifugation, and then the dried peptides were reconstituted in 0.5 M TEAB solution (pH 8.5).

According to the manufacturer’s protocol, proteins were labeled with an 8-plex iTRAQ Reagent Multiplex Kit (AB Sciex, USA). For labeling, one unit of iTRAQ reagent was thawed and reconstituted in 24 μL of isopropanol, and proteins were labeled with isobaric tags as follows: 113- and 114-tag for C1, 115- and 116-tag for C2, 117- and 118-tag for M200, and 119- and 121-tag for M1000. The labeled samples were incubated at 25 °C for 1 h, and the reaction was stopped with 100 μL of ddH_2_O. Then, the differentially labeled peptide mixtures were pooled and dried by vacuum centrifugation.

### 4.8. NanoLC-MS/MS Analysis

Pooled iTRAQ-labeled peptide mixtures were reconstituted with 100 μL of buffer A (2% acetonitrile, 20 mM NH_4_FA, pH 10.0, adjusted with NH_3_·H_2_O), loaded onto a Durashell C18 column (4.6 × 250 mm, 5 μm, 100 Å, Agela), and fractionated using a high performance liquid chromatography (HPLC) system (Thermo Dionex Ultimate 3000 BioRS). Buffer A and buffer B (80% acetonitrile, 20 mM NH_4_FA, pH 10.0, adjusted with NH_3_·H_2_O) were used to develop a gradient elution. Peptides were eluted at a flow rate of 1 mL/min with a gradient of 5% B in 7 min, 25% B in 16 min, and 5% B in 25 min. Elution was monitored by measuring the absorbance at 214 nm. A total of 12 fractions were collected at 1-min interval, desalted with a C18 column (Strata-X, Phenomenex) and dried in a vacuum concentrator for subsequent analysis.

The fractions were reconstituted in buffer A (0.1% formic acid, 5% acetonitrile), and separated by a nano LC-MS/MS system connected to a Q-exactive HF-X mass spectrometer (Thermo Fisher Scientific, USA). Peptides were loaded onto the eksigent Chromxp Trap Column (C18-CL, 350 μm × 0.5 mm, 3 μm, 120 Å, AB, CA, USA) by an autosampler, with a flow rate of 10 μL/min for 5 min, and then eluted onto an analytical C18 column (75 μm inner diameter, 10 cm length, 3 μm, AB, CA, USA). The samples were eluted with a gradient of buffer B (0.1% formic acid, 95% acetonitrile) at a flow rate of 300 nL/min using the following elution program: 5% to 30% buffer B for 0 to 65 min, 30% to 50% buffer B for 65 to 70 min, 50% to 80% buffer B for 70 to 85 min, 80% to 5% buffer B for 85 to 90 min.

The mass spectrometer was operated in the data-dependent mode to switch automatically between MS and MS/MS acquisition, and data was acquired using an ion source gas 1 of 5 psi, curtain gas of 35 psi, ion spray voltage floating of 2.5 kV, and interface heater temperature of 150 °C. The MS1 scan spectra were collected in the range 350–1500 m/z for 250 milliseconds, and mass tolerance was 50 mDa; the MS2 spectra were collected in the range 100–1500 m/z for 100 milliseconds; and dynamic exclusion was set to 12 s.

### 4.9. Database Searching, Protein Identification, Annotation, and Functional Analysis

Raw data from MS/MS were analyzed by Proteome Discoverer 2.1 (AB, Foster City, CA, USA) using the Paragon algorithm and ProGroup algorithm to perform search engine. Due to the unavailable database for proteins of *Avena sativa*, raw files were searched against a Uniprot protein database containing all plants (downloaded on 20171220, 2304711 protein sequences). For protein identification, the following parameters were set: precursor ion mass tolerance ± 10 ppm; fragment ion mass tolerance ± 0.02 Da, maximum missed cleavages for trypsin digestion were set to two, carbamidomethyl (C) and iTRAQ8plex (N-terminal, K) were set as fixed modifications, and oxidation (M) and acetyl (N-terminal) were set as dynamic modifications. Peptides with a false discovery rate (FDR) of 0.01 and at least one unique peptide included for iTRAQ labeling quantification were selected for further analysis. The DEPs were identified on the basis of FC and *p*-value calculated through T-test of each protein, performed using Proteome Discoverer 2.1. In this study, identified proteins with FC ≥ 1.5 (or ≤0.67) and *p*-value ≤ 0.05 were considered as DEPs.

The DEPs were used for COG (cluster of orthologous groups of proteins) category and annotation. GO (gene ontology) annotation of DEPs, including the biological process, cellular component, and molecular function, was performed using Uniprot (http://www.uniprot.org/). KEGG (Kyoto Encyclopedia of Genes and Genomes) database (http://www.genome.jp/kegg/pathway.html) was used to predict the main metabolic pathways.

GO enrichment and KEGG pathway analysis of the DEPs were performed, using the hypergeometric test to calculate the *p*-value of each GO term and KEGG pathway, and a *p*-value < 0.05 was identified as the threshold to determine the significant enrichment of differential proteins.

### 4.10. RNA Extraction and qRT-PCR

Total RNA from embryos was extracted using a TRNzol Kit (Tiangen Biotech, Beijing, China), according to the manufacturer’s instructions. The quantity of total RNA was confirmed by NanoDrop^®^ ND-2000 (Thermo Fisher Scientific, USA) and 1.0% denatured agarose gel electrophoresis. cDNA was synthesized using the PrimeScript^™^ RT reagent Kit with gDNA Eraser (Takara, Dalian, China). The qRT-PCR was performed using the SYBR^®^ Premix Ex Taq^™^ II (Tli RNaseH Plus) (Takara, Dalian, China), following the manufacturer’s instructions, with the ABI7900 Real-Time PCR thermal cycler (Applied Biosystems). Procedure of cycle conditions was as follows: 95 °C for 30 s, then 40 cycles at 95 °C for 5 s and 60 °C for 40 s, the final cycle of 95 °C for 10 s, 60 °C for 60 s, and 95 °C for 15 s. Gene *ACTIN2* was selected as the reference gene to normalize the expression level of target genes, which were calculated using the 2^−∆∆*C*t^ method. qRT-PCR determination was performed in three biological replicates and three technical replicates. The primer sequences used in this study are listed in Table S7.

### 4.11. Statistical Analysis

Results were presented as the means ± SE. Statistical analyses of the data were performed using one-way ANOVA test and the Duncan’s test was conducted to compare the means of different treatments at the *p* < 0.05 level. All analyses were performed by SPSS Statistics software (version 17.0).

## 5. Conclusions

**Figure 8:**
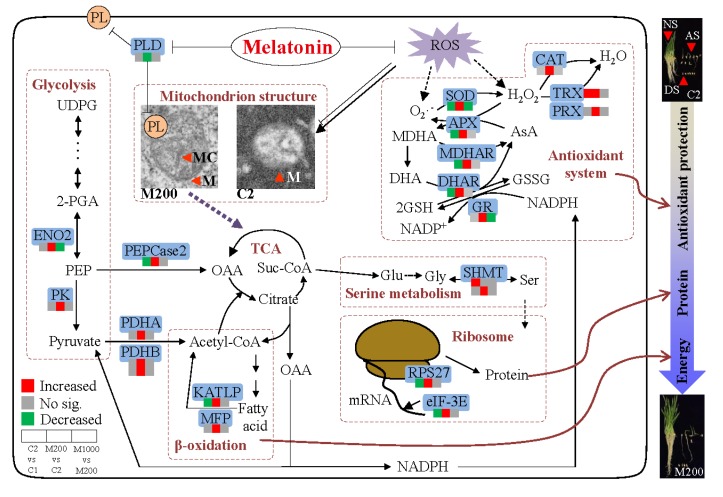
Hypothetic schemata deciphering the physiological and molecular mechanisms related to melatonin priming on germination in aged oat seeds. PL, phospholipid; Suc-CoA, succinate CoA; Glu, glutamate; Gly, glycine; Ser, serine.

On the basis of the conjoint analysis of ultrastructure, physiology, and proteomics, we confirmed a putative schematic pathway that could perform during melatonin-mediated germination in aged oat seeds (Figure 8). To summarize, three major events were involved in these results. Melatonin repaired cell ultrastructure through the inhibition of PLD expression and phospholipid degradation which, in turn, improved the development of mitochondria and cytomembrane, and therefore vigor level in aged oat seeds was enhanced and germination and seedling growth, to some extent, could be recovered. The more complete cellular ultrastructure was conducive to help melatonin play its antioxidant protective role through the enhanced antioxidant capacity in the AsA-GSH cycle and the reduction of ROS accumulation. Furthermore, the DEPs related to energy supply (PEPCase2, KATLP) and protein translation (RPS27, eIF-3E) were significantly altered in response to melatonin priming in aged oat seeds, in particular, the KATLP and its involved β-oxidation pathway could provide a more adequate energy supply for maintaining TCA cycle stability and protein synthesis. Overall, it could be clearly demonstrated that the above major events were involved in germination and seedling growth and they provided new insights into the underlying melatonin regulatory mechanisms that occurred in embryos of aged oat seeds.

## Figures and Tables

**Table 1:** qRT-PCR analysis of selected DEPs in embryo of oat seeds under aging and melatonin priming.

Compared Samples	Accession	Description	FC of DEPs	FC in qRT-PCR
C2 vs. C1	W5DQ12	Proteasome subunit beta type	0.093	0.497 ± 0.082
	B6TJX6	PTI1-like tyrosine-protein kinase 3	0.057	6.306 ± 0.812
	A0A0D3GV84	Lon protease homolog, mitochondrial	0.065	2.413 ± 0.472
	A0A1Y1HNC9	Eukaryotic translation initiation factor eIF-4A	15.348	2.312 ± 0.268
M200 vs. C2	A9UIF0	Phospholipase D	0.089	1.197 ± 0.040
	A0A1D5RZK8	Nascent polypeptide-associated complex subunit beta	7.442	1.749 ± 0.143
	Q10P35	Enolase 2, putative, expressed	7.780	2.061 ± 0.063
	M1Q6S1	Starch synthase, chloroplastic/amyloplastic	4.453	0.279 ± 0.070
	W5AA91	Importin subunit alpha	5.507	15.181 ± 1.293
M1000 vs. C2	A0A072VAH0	Peroxidase	25.234	0.591 ± 0.093
	A0A1D5RZK8	Nascent polypeptide-associated complex subunit beta	6.870	1.808 ± 0.206
	A0A1D5VEP2	Obg-like ATPase 1	0.106	2.596 ± 0.309
	F2DCZ4	Aconitate hydratase	0.240	1.590 ± 0.076
	A0A1D5RU17	Guanosine nucleotide diphosphate dissociation inhibitor	0.061	61.430 ± 12.310
M1000 vs. M200	A0A072VAH0	Peroxidase	15.909	0.873 ± 0.113
	A9UIF0	Phospholipase D	5.824	0.668 ± 0.054
	Q10P35	Enolase 2, putative, expressed	0.293	0.888 ± 0.049
	M1Q6S1	Starch synthase, chloroplastic/amyloplastic	0.125	2.267 ± 0.585
	A0A1D5VEP2	Obg-like ATPase 1	0.100	0.686 ± 0.059
	F2DCZ4	Aconitate hydratase	0.049	1.068 ± 0.011

Note: FC means fold change.

## Supplementary Materials

Supplementary materials can be found at https://www.mdpi.com/1422-0067/21/5/1898/s1.

## Abbreviations

APX	Ascorbate peroxidase
AsA-GSH	Ascorbate-glutathione
CAT	Catalase
DHAR	Dehydroascorbate reductase
GI	Germination index
GP	Germination percentage
GR	Glutathione reductase
H_2_O_2_	Hydrogen peroxide
iTRAQ	Isobaric tags for relative and absolute quantification
KATLP	3-Ketoacyl-CoA thiolase-like protein
LPO	Lipid peroxidation
MDA	Malondialdehyde
MDHAR	Monodehydroascorbate reductase
O_2_^−^·	Superoxide anion
OH·	Hydroxyl radical
ROS	Reactive oxygen species
SL	Shoot length
SOD	Superoxide dismutase
SVI	Seedling vigor index
SW	Shoot weight
TEM	Transmission electron microscopy
