# Supplementary material for: Melatonin Priming Alleviates Aging-Induced Germination Inhibition by Regulating β-oxidation, Protein Translation, and Antioxidant Metabolism in Oat (Avena sativa L.) Seeds
Source: Int J Mol Sci. 2020 Mar 10;21(5):1898. doi: 10.3390/ijms21051898 (PMC7084597; doi:10.3390/ijms21051898)
Supplement: Supplementary file 1 [file ijms-21-01898-s001.zip › Supplementary Materials/Legends of supplementary materials.docx]

## Legends of supplementary files

Figure S1. Changes in germination and imbibition of oat seeds.

Figure S2. Function annotation of the total identified proteins in oat’s embryos under aging and melatonin priming.

Table S1. Total identified proteins by the iTRAQ-based quantitative proteome.

Table S2. The DEPs quantified in embryo of oat seeds under aging process.

Table S3. The DEPs quantified in embryo of oat seeds under melatonin priming process.

Table S4. Totally 70 DEPs were shared by aging and melatonin priming processes and their different expression profiles.

Table S5. The DEPs with annotated function and their expressed information assigned to different categories in oat’s embryo during aging and melatonin priming.

Table S6. The TOP10 KEGG pathways of DEPs in oat’s embryo under aging and melatonin priming.

Table S7. Primer sequences of qRT-PCR in this study.
