# Supplementary material for: Melatonin Priming Alleviates Aging-Induced Germination Inhibition by Regulating β-oxidation, Protein Translation, and Antioxidant Metabolism in Oat (Avena sativa L.) Seeds
Source: Int J Mol Sci. 2020 Mar 10;21(5):1898. doi: 10.3390/ijms21051898 (PMC7084597; doi:10.3390/ijms21051898)
Supplement: Supplementary file 1 [file ijms-21-01898-s001.zip › Supplementary Materials/Supplementary Figures.docx]

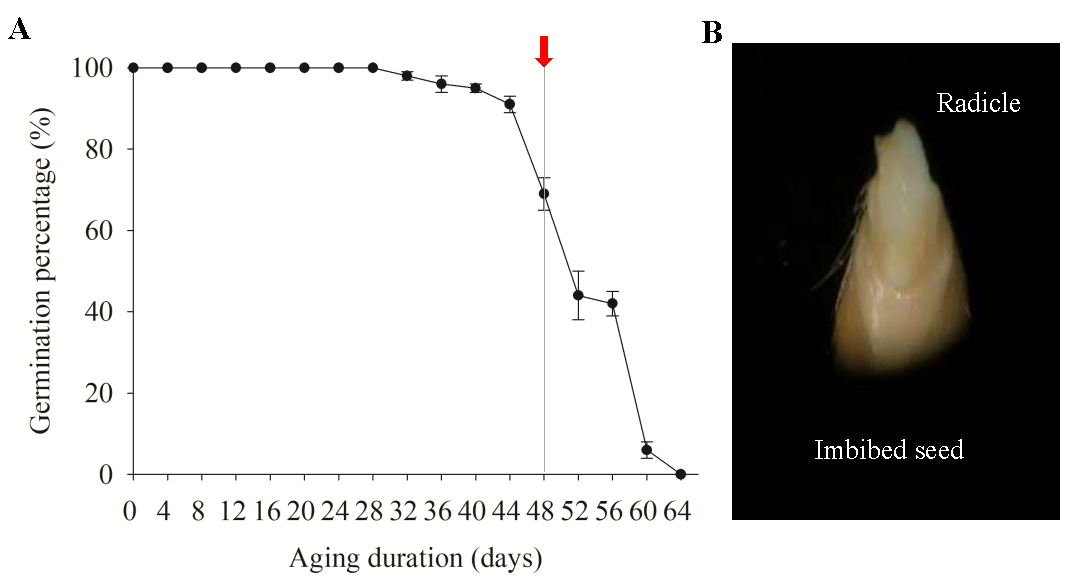


**Figure S1.** Changes in germination and imbibition of oat seeds. (**A**) A reverse ‘S-shaped’ curve of germination percentage (GP) during aging process. Oat seeds with 10% moisture content in bags (25 g/bag) were incubated in an electric thermostatic cistern at 45°C, and regularly collected at 4 days’ intervals. Afterward, germination test for aged seeds were performed as described in ‘Materials and Methods 4.3.’, and GP was calculated. Finally, the GP curve with aging duration prolonging was plotted. (**B**) Embryo status used to collected samples after imbibition with distilled water for 12 h at 20°C in the dark.


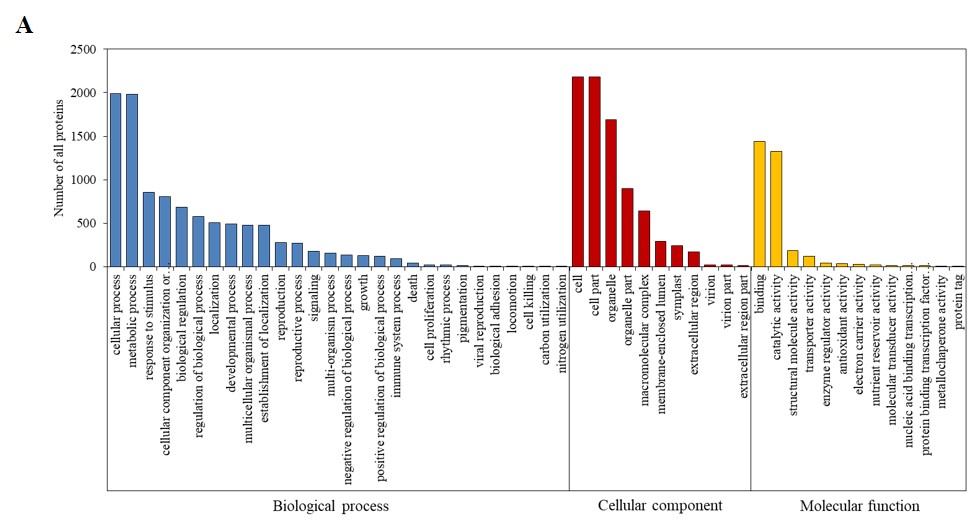


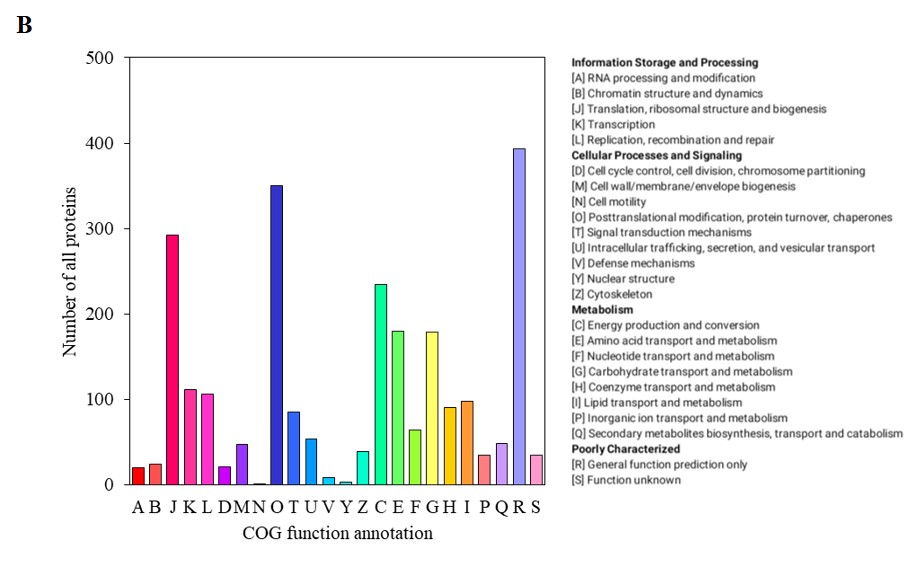


**Figure S2.** Function annotation of the total identified proteins in oat’s embryos under aging and melatonin priming. **(A)** GO annotation. **(B)** COG function annotation.
